# Supplementary material for: Exact solution of three dimensional schrödinger equation with power function superposition potential
Source: PLoS One. 2023 Nov 28;18(11):e0294851. doi: 10.1371/journal.pone.0294851 (PMC10684078; doi:10.1371/journal.pone.0294851)
Supplement: S1 Dataset — (DOCX) [file pone.0294851.s001.docx]

# Minimal Data Set

**Table 1 Reduced masses and spectroscopically determined properties of various diatomic molecules in the ground electronic state**

| Parameter | O_2_ | HCl | CO |
| --- | --- | --- | --- |
| *D_e_* (in eV) | 5.156658828 | 4.619061175 | 10.84514471 |
| *r_e_* (in nm) | 0.1208 | 0.12746 | 0.11282 |
| *µ* (in amu) | 7.997457504 | 0.9801045 | 6.860586 |

**Table 2 The coefficients of the potential**$V(r)=a_{-2}r^{-2}+a_{-1}r^{-1}+a_{0}$

| coefficient | O_2_ | HCl | CO |
| --- | --- | --- | --- |
| $a_{-2}$(in eV nm^2^) | 0.075249266 | 0.075041506 | 0.138040824 |
| $a_{-1}$(in eV nm) | -1.245848773 | -1.177491075 | -2.447098452 |
| $a_{0}$ | 0 | 0 | 0 |

**Table 3 The coefficients of the potential**$V(r)=a_{-2}r^{-2}+a_{0}+a_{2}r^{2}$

| coefficient | O_2_ | HCl | CO |
| --- | --- | --- | --- |
| $a_{-2}$(in eV nm^2^) | 0.075249266 | 0.075041506 | 0.138040824 |
| $a_{2}$(in eV/ nm^2^) | 353.3739493 | 284.3190019 | 852.0462326 |
| $a_{0}$(in eV) | -15.46997648 | -13.85718353 | -32.53543413 |

**Table 4 Energy eigenvalues (in eV) for the various *n_r_* and *l* quantum numbers for a few diatomic molecules by using Eq. (26), where ,$e=1.602176634\times10^{-19}C$,$1amu=1.66053906660\times10^{-27}kg$ (from SI Brochure 9th edition of the SI Brochure, available on the BIPM web page: www.bipm.org)**

| *n_r_* | *l* | O_2_/eV | HCl/eV | CO/eV |
| --- | --- | --- | --- | --- |
| 0 | 0 | -5.126358800 | -4.541848670 | -10.79431563 |
| 1 | 0 | -5.066641679 | -4.393729259 | -10.69384082 |
| 1 | 1 | -5.066292858 | -4.391295181 | -10.69337213 |
| 2 | 0 | -5.007961982 | -4.252739178 | -10.59476237 |
| 2 | 1 | -5.007619203 | -4.250421300 | -10.59430017 |
| 2 | 2 | -5.006933786 | -4.245793196 | -10.59337591 |
| 3 | 0 | -4.950295818 | -4.118428126 | -10.49705450 |
| 3 | 1 | -4.949958943 | -4.116219168 | -10.49659869 |
| 3 | 2 | -4.949285330 | -4.111808458 | -10.49568718 |
| 3 | 3 | -4.948275256 | -4.105210348 | -10.49432021 |
| 4 | 0 | -4.893619980 | -3.990380802 | -10.40069206 |
| 4 | 1 | -4.893288873 | -3.988274045 | -10.40024251 |
| 4 | 2 | -4.892626794 | -3.984067324 | -10.39934352 |
| 4 | 3 | -4.891634014 | -3.977774169 | -10.39799534 |
| 4 | 4 | -4.890310938 | -3.969414739 | -10.39619830 |
| 5 | 0 | -4.837911919 | -3.868213688 | -10.30565046 |
| 5 | 1 | -4.837586450 | -3.866202923 | -10.30520706 |
| 5 | 2 | -4.836935644 | -3.862187803 | -10.30432036 |
| 5 | 3 | -4.835959766 | -3.856181097 | -10.30299061 |
| 5 | 4 | -4.834659212 | -3.848201827 | -10.30121815 |
| 5 | 5 | -4.833034511 | -3.838275121 | -10.29900343 |

**Table 5 Energy eigenvalues (in eV) for the various *n_r_* and *l* quantum numbers for a few diatomic molecules by using Eq. (36), where ,$e=1.602176634\times10^{-19}C$,$1amu=1.66053906660\times10^{-27}kg$ (from SI Brochure 9th edition of the SI Brochure, available on the BIPM web page: www.bipm.org)**

| *n_r_* | *l* | O_2_/eV | HCl/eV | CO/eV |
| --- | --- | --- | --- | --- |
| 0 | 0 | -5.095835179 | -4.463000904 | -10.74318765 |
| 1 | 0 | -4.974277415 | -4.151536686 | -10.53939319 |
| 1 | 1 | -4.973919238 | -4.148911890 | -10.53891450 |
| 2 | 0 | -4.852719651 | -3.840072468 | -10.33559872 |
| 2 | 1 | -4.852361474 | -3.837447672 | -10.33512004 |
| 2 | 2 | -4.851645158 | -3.832200316 | -10.33416269 |
| 3 | 0 | -4.731161887 | -3.528608250 | -10.13180426 |
| 3 | 1 | -4.730803710 | -3.525983454 | -10.13132557 |
| 3 | 2 | -4.730087394 | -3.520736098 | -10.13036823 |
| 3 | 3 | -4.729013012 | -3.512870643 | -10.12893229 |
| 4 | 0 | -4.609604123 | -3.217144032 | -9.928009797 |
| 4 | 1 | -4.609245946 | -3.214519236 | -9.927531108 |
| 4 | 2 | -4.608529630 | -3.209271880 | -9.926573761 |
| 4 | 3 | -4.607455248 | -3.201406425 | -9.925137821 |
| 4 | 4 | -4.606022914 | -3.190929539 | -9.923223382 |
| 5 | 0 | -4.488046359 | -2.905679814 | -9.724215333 |
| 5 | 1 | -4.487688182 | -2.903055018 | -9.723736644 |
| 5 | 2 | -4.486971866 | -2.897807662 | -9.722779297 |
| 5 | 3 | -4.485897484 | -2.889942207 | -9.721343357 |
| 5 | 4 | -4.484465150 | -2.879465321 | -9.719428918 |
| 5 | 5 | -4.482675011 | -2.866385850 | -9.717036107 |

**Table 6 Difference (in eV) of energy eigenvalues calculated by Eq. (26) and Eq. (36)**

| *n_r_* | *l* | Difference for O_2_ | Difference for HCl | Difference for CO |
| --- | --- | --- | --- | --- |
| 0 | 0 | 0.030523621 | 0.078847766 | 0.05112798 |
| 1 | 0 | 0.092364264 | 0.242192573 | 0.15444763 |
| 1 | 1 | 0.092373620 | 0.242383291 | 0.15445763 |
| 2 | 0 | 0.155242331 | 0.412666710 | 0.25916365 |
| 2 | 1 | 0.155257729 | 0.412973628 | 0.25918013 |
| 2 | 2 | 0.155288628 | 0.413592880 | 0.25921322 |
| 3 | 0 | 0.219133931 | 0.589819876 | 0.36525024 |
| 3 | 1 | 0.219155233 | 0.590235714 | 0.36527312 |
| 3 | 2 | 0.219197936 | 0.591072360 | 0.36531895 |
| 3 | 3 | 0.219262244 | 0.592339705 | 0.36538792 |
| 4 | 0 | 0.284015857 | 0.773236770 | 0.472682263 |
| 4 | 1 | 0.284042927 | 0.773754809 | 0.472711402 |
| 4 | 2 | 0.284097164 | 0.774795444 | 0.472769759 |
| 4 | 3 | 0.284178766 | 0.776367744 | 0.472857519 |
| 4 | 4 | 0.284288024 | 0.778485200 | 0.472974918 |
| 5 | 0 | 0.349865560 | 0.962533874 | 0.581435127 |
| 5 | 1 | 0.349898268 | 0.963147905 | 0.581470416 |
| 5 | 2 | 0.349963778 | 0.964380141 | 0.581541063 |
| 5 | 3 | 0.350062282 | 0.966238890 | 0.581647253 |
| 5 | 4 | 0.350194062 | 0.968736506 | 0.581789232 |
| 5 | 5 | 0.350359500 | 0.971889271 | 0.581967323 |

**Table 7 Comparison of the energy levels (in eV) for the various *n_r_* and *l* quantum numbers for diatomic molecule O_2_ calculated using different methods.**

| *n_r_* | *l* | O_2_(by Eq. 26) | O_2_(by Eq. 36) | O_2_(by AIM) | O_2_(by EQR) |
| --- | --- | --- | --- | --- | --- |
| 0 | 0 | -5.126358800 | -5.095835179 | −5.126358625 | −5.126358620071 |
| 1 | 0 | -5.066641679 | -4.974277415 | −5.066641151 | −5.066641146718 |
| 1 | 1 | -5.066292858 | -4.973919238 | −5.066292323 | −5.066292321402 |
| 2 | 0 | -5.007961982 | -4.852719651 | −5.007961116 | −5.007961110233 |
| 2 | 1 | -5.007619203 | -4.852361474 | −5.007618329 | −5.007618327191 |
| 2 | 2 | -5.006933786 | -4.851645158 | −5.006932904 | −5.006932902380 |
| 3 | 0 | -4.950295818 | -4.731161887 | −4.950294624 | −4.950294618656 |
| 3 | 1 | -4.949958943 | -4.730803710 | −4.949957740 | −4.949957739138 |
| 3 | 2 | -4.949285330 | -4.730087394 | −4.949284119 | −4.949284118344 |
| 3 | 3 | -4.948275256 | -4.729013012 | −4.948274034 | −4.948274032620 |
| 4 | 0 | -4.893619980 | -4.609604123 | −4.893618469 | −4.893618463868 |
| 4 | 1 | -4.893288873 | -4.609245946 | −4.893287355 | −4.893287353086 |
| 4 | 2 | -4.892626794 | -4.608529630 | −4.892625268 | −4.892625266816 |
| 4 | 3 | -4.891634014 | -4.607455248 | −4.891632476 | −4.891632475505 |
| 4 | 4 | -4.890310938 | -4.606022914 | −4.890309388 | −4.890309384483 |
| 5 | 0 | -4.837911919 | -4.488046359 | −4.837910103 | −4.837910098245 |
| 5 | 1 | -4.837586450 | -4.487688182 | −4.837584627 | −4.837584625235 |
| 5 | 2 | -4.836935644 | -4.486971866 | −4.836933812 | −4.836933811639 |
| 5 | 3 | -4.835959766 | -4.485897484 | −4.835957923 | −4.835957922172 |
| 5 | 4 | -4.834659212 | -4.484465150 | −4.834657357 | −4.834657353568 |
| 5 | 5 | -4.833034511 | -4.482675011 | −4.833032637 | −4.833032634174 |
