# Supplementary material for: Exact solution of three dimensional schrödinger equation with power function superposition potential
Source: PLoS One. 2023 Nov 28;18(11):e0294851. doi: 10.1371/journal.pone.0294851 (PMC10684078; doi:10.1371/journal.pone.0294851)
Supplement: Attachment — Submitted filename: Report on PONE-D-23-15630.pdf [file pone.0294851.s002.pdf]

Report on PONE-D-23-15630 manuscript entitled: Exact Solution of Three-Dimensional Schrödinger Equation with Power Function Superposition Potential

General comments

- In this manuscript the authors' purported objective is to develop an *analytic solution* to the three-dimensional Schrodinger equation using the power function superposition potential. To this end they considered two forms of such potentials as given by Eqns. (13) and (14) in the manuscript.
- The eigenvalues for various radial and angular momenta are then obtained using the derived analytical solutions of the Schrodinger equation (Eqns. 26 and 36) for a few diatomic molecules.

Typos / language

- It is noted that in several places after the equation, continuation lines start with a capital letter instead of a small letter. For example, the sentence after Eqn (1) starts with "Where" instead of "where. Similar occurrences appear **after**
    - Eqn (3)
    - Eqn (4)
    - Table 1
    - Eqn (17)
    - Eqn (25)
    - Eqn (27)
    - Eqn (35)
