# Supplementary material for: Exact solution of three dimensional schrödinger equation with power function superposition potential
Source: PLoS One. 2023 Nov 28;18(11):e0294851. doi: 10.1371/journal.pone.0294851 (PMC10684078; doi:10.1371/journal.pone.0294851)
Supplement: Attachment — Submitted filename: Report on PONE-D-23-15630R1.pdf [file pone.0294851.s004.pdf]

Report on PONE-D-23-15630R1 revised manuscript entitled: Exact Solution of Three-Dimensional Schrödinger Equation with Power Function Superposition Potential

General comment

- The authors have addressed all the suggested changes and recommendations, including the typos.
