# Supplementary material for: Exact solution of three dimensional schrödinger equation with power function superposition potential
Source: PLoS One. 2023 Nov 28;18(11):e0294851. doi: 10.1371/journal.pone.0294851 (PMC10684078; doi:10.1371/journal.pone.0294851)
Supplement: Attachment — Submitted filename: Response to Reviewers.docx [file pone.0294851.s003.docx]

**A reply letter to the reviewer**

Dear Reviewer,

Thank you very much for your review. Your review comments were very relevant and I was deeply inspired after reading them.

For the following questions, our response is as follows:

1. Is the manuscript technically sound, and do the data support the conclusions?

In our manuscript, the general form of the power function superposition potential is used to construct two different power function superposition potentials with exact analytical solutions, which is based on the supersymmetric quantum mechanics. The exact bound state energy eigenvalues of several different diatomic molecules are calculated for any *l* angular momentum quantum number bound by these two exactly solvable potential.

The manuscript must describe a technically sound piece of scientific research with data that supports the conclusion, which is the creed we adhere to when conducting research.

2. Has the statistical analysis been performed appropriately and rigorously?

Since we construct potential functions with exact analytical solutions and calculate their energy levels, there is no need for statistical analysis. The results provided by the reviewer are correct.

3. Have the authors made all data underlying the findings in their manuscript fully available?
We have provided all the data underlying the findings in our manuscript fully available.

4. Is the manuscript presented in an intelligible fashion and written in standard English?

Our manuscript is presented in an intelligible fashion and written in standard English. Thank you for the reviewer's comments.

5. Review Comments to the Author

We have already introduced in our manuscript how to obtain the number of coefficient constants for the potentials. The original text is as follows:

When $r=r_{e}$, $V(r)=-D_{e}$ and $\frac{dV(r)}{dr}=0$, the coefficient of the potential function $V(r)$ can be obtained through calculation.

If $V(r)=a_{-2}r^{-2}+a_{-1}r^{-1}+a_{0}$, it can be obtained that $a_{-2}=D_{e}r_{e}^{2}$, $a_{-1}=-2D_{e}r_{e}$, $a_{0}=0$.

If $V(r)=a_{-2}r^{-2}+a_{0}+a_{2}r^{2}$, it can be known that $a_{-2}=D_{e}r_{e}^{2}$, $a_{2}=\frac{D_{e}}{r_{e}^{2}}$, $a_{0}=-3D_{e}$.

We did not list the number of coefficient constants for these potentials in our manuscript, and we have added them in the revised manuscript.

Regarding the problem in Table 4, we did compare the results of two different potentials, and we hope to obtain the result that as the quantum number increases, the influence of the harmonic oscillator potential exceeds that of the Coulomb potential. The results obtained were not compared with experimental data or other methods.

In the revised manuscript, we have made modifications and compared our results with other methods to obtain better results.

There are indeed several issues with uppercase letters in our manuscript, and we have made modifications.

We have made improvements to the issues in the introduction and references.

Kind regards,

Meihuan Fu
